# Effects of Different Backpack Loads in Acceleration Transmission during Recreational Distance Walking

**DOI:** 10.2478/hukin-2013-0028

**Published:** 2013-07-05

**Authors:** Angel G. Lucas-Cuevas, Pedro Pérez-Soriano, Michael Bush, Aaron Crossman, Salvador Llana, Juan M. Cortell-Tormo, José A. Pérez-Turpin

**Affiliations:** 1 Department of Sport and Physical Education, University of Valencia, Spain.; 2 Department of General and Specific Didactics, University of Alicante, Spain. .

**Keywords:** accelerometry, load carriage, treadmill, walking

## Abstract

It is well established nowadays the benefits that physical activity can have on the health of individuals. Walking is considered a fundamental method of movement and using a backpack is a common and economical manner of carrying load weight. Nevertheless, the shock wave produced by the impact forces when carrying a backpack can have detrimental effects on health status. Therefore, the aim of this study was to investigate differences in the accelerations placed on males and females whilst carrying different loads when walking. Twenty nine sports science students (16 males and 13 females) participated in the study under 3 different conditions: no weight, 10% and 20% body weight (BW) added in a backpack. Accelerometers were attached to the right shank and the centre of the forehead. Results showed that males have lower accelerations than females both in the head (2.62 ± 0.43G compared to 2.83 + 0.47G) and shank (1.37 ± 0.14G compared to 1.52 ± 0.15G; p<0.01). Accelerations for males and females were consistent throughout each backpack condition (p>0.05). The body acts as a natural shock absorber, reducing the amount of force that transmits through the body between the foot (impact point) and head. Anthropometric and body mass distribution differences between males and females may result in women receiving greater impact acceleration compared to men when the same load is carried.

## Introduction

Nowadays, it is generally said and stated that physical activity is good for human health ( Griera et al., 2007 ). It has been concluded that doing intermediate to high intensity aerobic activity five times a week for 30 minutes is enough to benefit from several of the positive effects that accompany physical exercise such as decreasing arterial pressure and resting heart rate, maintaining and losing weight, strengthening ligaments and tendons, increasing joint mobility and bone mass density, along with decreasing the risk of suffering from diseases such as obesity, hypertension or arteriosclerosis, among many others ( Garber et al., 2011 ; Taaffe et al., 1997 ; Voloshin, 2000 ; Wilmore et al., 2008 ).

However, there is an increasing current concern regarding the problems originated in the back related to physical exercise activities, from children carrying excessive load backpacks to school to adults dealing with too heavy working loads. Many researchers have been carrying out studies to identify the potential harm that overweighting load activities can produce on the human body ( Holt et al., 2005 ; Lafortune et al., 1996 ; Taaffe et al., 1997 ; Voloshin, 2000 ).

It is necessary to bear in mind that not only labour tasks (i.e. construction or military manoeuvres) may lead to injury and body pain ( Birrel et al., 2007 ), but also recreational and leisure activities such as hiking ( Forjuoh et al., 2004 ; Voloshin, 2000 ) or students walking to school carrying heavy backpacks full of books, after-school activity supplies (music and sports equipment) and personal objects ( Bauer and Freivalds, 2009 ; Chiang et al., 2006 ; Lockhart et al., 2004 ).

Research looking into body pain as a consequence of carrying loads underlies factors such as the amount of load, time spent carrying and repeated loading, position and way of carrying the load, design of the backpack, physical condition as well as physiological characteristics such as age and gender of the individual ( Chansirinukor et al., 2001 ; Golriz and Walker, 2012 ; Grimmer and Williams, 2000 ; Keller et al., 1996 ; Knapik et al., 1996 ; Lockhart et al., 2004 ; Voloshin, 2000 ). Taking into account that a person may take over 6000 steps per day on average, making a cumulative of 2.5 million steps per year ( Voloshin, 2000 ), it becomes highly recommended to control the conditions under which the load is carried and its subsequent consequences for the human body.

Studying the physical and mechanical behaviour of the body when moving, every time a body segment (such as the foot) contacts with a rigid surface (such as the ground), an impact force is produced, generating a shock wave that travels through the musculoskeletal system from the foot to the head ( Lafortune et al., 1996 ). The joints of the human musculoskeletal systems act like a shock absorber, which means that they attenuate and dissipate the shock initiated from the foot, protecting the joints located further along the path of the shock wave propagation towards the head (Wosk and Voloshin, 1981; Voloshin, 2000 ). In order to measure these shock waves, an accelerometer is commonly attached to a body part where the bone is very close to the skin such as the tibial tuberosity, the sacrum or the forehead to measure the shock waves experienced during any physical activity ( Harman et al., 2000 ; Holt et al., 2005 ; Kiiski et al., 2008 ; Pérez and Llana, 2007 ; Ren et al., 2007 ).

Regarding the effect of these shocks over the human body, there are two main contrary views. On the one hand, it is very well documented that a bone decreases in thickness and density as a direct response to a decrease in loading ( Voloshin, 2000 ). On the other hand, these shocks, when produced during a long period of time (such as a marathon race) or under uncontrolled parameters (impairment of the musculoskeletal system, overweight backpack carriage), are considered an important factor in the development of spinal injuries and degenerative changes in joint and articular cartilage ( Lafortune et al., 1996 ). Excess dynamic loading on the human musculoskeletal system may lead to the development of a variety of musculoskeletal disorders such as osteoarthritis or bone stress fractures, turning into muscular aches, back strain, bad posture and low back pain ( Voloshin, 2000 ). Specifically in student population carrying school backpacks (period when musculoskeletal system and spinal experiment their biggest growth), heavy carriage has been stated as a significant contributing risk factor for neck, shoulder and back pain (both high and low back), fatigue, muscle soreness, numbness, discomfort, stress fractures of the tibia and knee joint problems ( Birrel et al., 2007 ; Chiang et al., 2006 ; Golriz and Walker, 2011 ; Hong and Cheung, 2003 ; Lockhart et al., 2004 ).

Aiming to avoid carrying excessive weight and prevent aforementioned injuries, experts recommend that school backpacks should not exceed 10–15% of the individual’s body mass ( Furjuoh et al., 2004 ; Hong and Li, 2005 ; Lindstrom-Hazel, 2009 ). Regarding the adult population, the recommended lifting limit in the United States is 23 kg, whereas in Italy there is a restriction by law of 30 kg and 20 kg maximum load allowed to be lifted during work for males and females, respectively ( Negrini et al., 1999 ), although literature within adult subjects is scarce and, therefore, further investigation is necessary.

Research investigating backpack load has not paid attention on how the gender may affect the shock wave propagation along the body, but focused mainly on the relationship between backpack loads and pain within student population, providing inconclusive results. While some studies report a greater number of females experiencing pain for the same backpack weight ( Golriz and Walker, 2011 ; Korovessis et al., 2004 ; Moore et al., 2007 ; Navuluri and Navuluri, 2006 ; Troussier et al., 1994 ), other authors report these figures to be dependant on the different peak growth rate between males and females during those years and, therefore, found no differences between males and females when carrying a given load ( Cavallo et al., 2003 ; Grimmer and Williams, 2000 ; Young et al., 2006 ). Therefore, the aim of this study was to examine the behavior of the vertical acceleration experienced both at the head and the shank and note whether differences are found between males and females when carrying a backpack load.

## Material and Methods

### Participants

Twenty nine sports science students (16 males and 13 females) took part in the experiment ( Table 1 ). The University of Valencia ethics subcommittee approved the study and an informed consent and a health history questionnaire were signed by the participants.

### Experimental Design

Participant’s body mass and height were recorded and 10% BW and 20% BW was set aside for each participant. Participants warmed up “ad libitum” and familiarized with the treadmill for ten minutes in order to become accustomed to walking on the treadmill at a given speed and to make sure they did not have any discomfort. Each participant was required to walk at 1.3 m/s (4.68 km/h) on a Technogym treadmill (Excite Run 700, TechnogymSpA, Gambettola, Italy).

Participants were required to carry a back pack during each of the conditions in which the weight was added. In order for the results to be normalised and compared between individuals, participants wore the same brand and type of shoes. Two uniaxial accelerators (Signal Frame, SportMetrics, Valencia, Spain) were attached in the centre of the forehead and the tibia of the right shank. The accelerometers recorded samples at the rate of 100 Hz and had a maximum range of + 10 G. Each accelerometer had an independent receiver box which was connected to an independent computer (both computers were synchronised by using a digital signal (trigger) which allowed the investigators to start the acquisition of data at the same time). Once participants had become familiarised with the setup of the experiment, they were told to straddle the treadmill with the right foot first and the researcher began recording (Signal Frame, SportMetrics, Valencia, Spain). After recording 20–30 steps, participants were told to stop, at which point they straddled the treadmill in order for weight to be added or removed before the procedure repeated.

Three levels of backpack load were tested: 0% BW, 10% BW and 20% BW. Weights were added into a backpack placed on the participants back (Crestone 60, The North Face, Lugano, Italy) and a grace period of 5 minutes was provided in order to get comfortable at the different weight conditions. To negate learning effects, variable test conditions (back pack mass) were conducted with randomised controlled trials. All the way through the experiment participants were given verbal encouragement and were monitored closely throughout the trials.

#### Data Analysis

All data was recorded using Signal Frame ©Software. Each participant had three steps selected at random for each of the back pack masses. The first 5 seconds of data were disregarded as the participant was forming their normal gait after stepping on a moving treadmill. The average of the peak acceleration for those three steps measured in “G” was calculated for the shank and head and they were imported into SPSS v.19 (SPSS Inc., Chicago, Illinois) for statistical analysis. Male and female results were compared through independent samples t-tests, analysing the differences between shank and head separately. A One way ANOVA was used to analyse the effect of backpack weight on shank and head accelerations, where males and females head and shank results were analysed independently. Post hoc tests (Bonferroni), with alpha level set at p<0.05, were used to provide details as to the whereabouts of significant differences.

## Results

**Figure 1:**
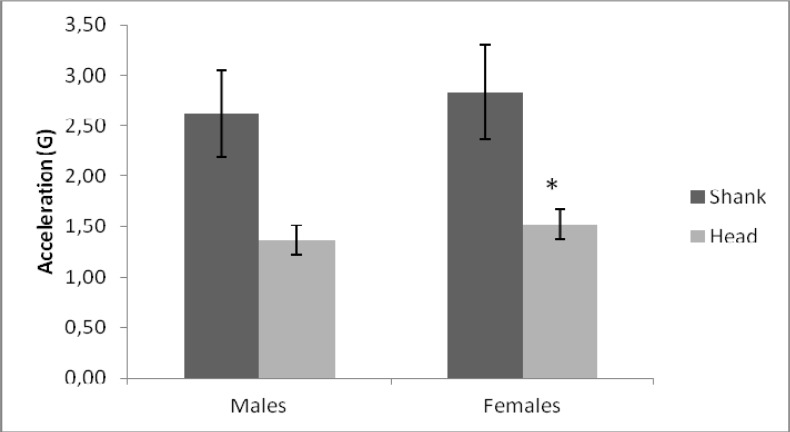
Accelerations measured in the head and the shank for males and females (*) p<0.01.

**Figure 2:**
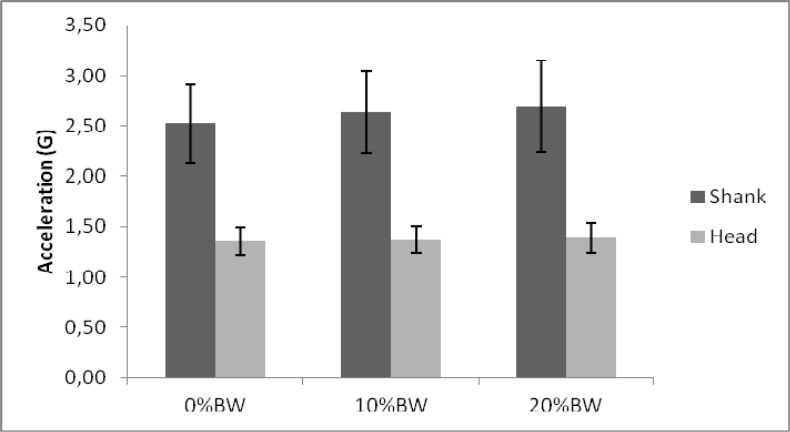
*
Male results for different backpack conditions
*

**Figure 3:**
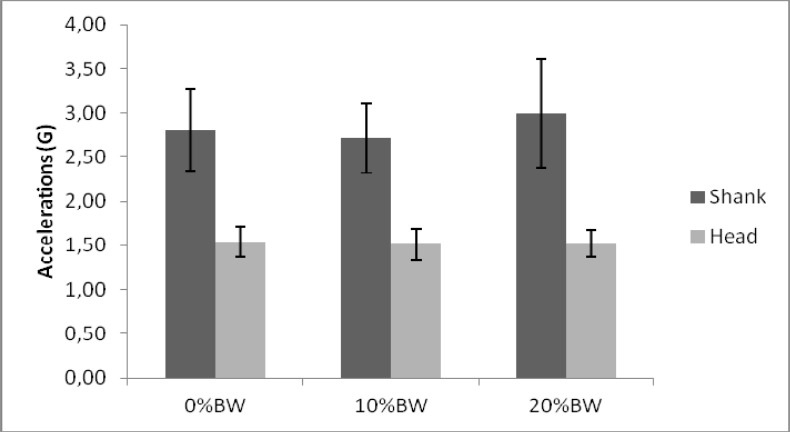
*
Female results for different backpack weights
*

Female’s accelerations were higher than males, both in the shank and at the head ( Figure 1 ). Further analysis was conducted by analysing accelerations at the shank and head when carrying different backpack loads ( Figure 2 and 3 ). Male accelerations in the shank increased as the load condition was heavier ( Figure 2 ). Although this increase was noted, no significant difference was found among conditions. At the head a similar increase was seen between the 3 conditions, with a minor increase across the different backpack weights. No significant differences were found between the accelerations in the 3 conditions measured at the head (p>0.05).

Although females were shown to have greater accelerations compared to males, when analysing the changes in different backpack weights, the females showed a consistent pattern across the conditions ( Figure 3 ). Although the highest acceleration at the head was observed in the 0% BW trial, the measured values were fairly consistent across the 3 trials. No significant differences were found either at the head or the shank between the 3 trials (p>0.05). The results of the females show higher accelerations at the head and shank compared to males, but also show that the spread of data is less consistent than their male counterparts.

## Discussion

The main aim of the study was to investigate the effects of different loads placed on the human body whilst walking, analysing the differences in the accelerations at the head and shank in both male and female individuals.

The results presented in the current study are in accordance with those observed in similar studies, even though most of the evidence is based on populations including males and females, studies aimed at identifying differences in impact accelerations between men and women are scarce. Perry et al. (1995) found that recreational male runners registered tibial acceleration values of 2.8 G when walking at 1.5 m/s whereas Rowlands and Stiles (2012) analysed impact accelerations during a variety of physical activities to compare the validity of an accelerometer attached to the wrist and to the waist. These authors registered similar peak accelerations using both types of devices and obtained values between 1.5 G and 2.5 G during slow and fast walking respectively.

On the other hand, Henriksen et al. (2008) did take into account the gender of the participants in their study although they did not measure the effect of carrying a backpack. Similar to the results observed in the present study, these authors also found greater peak tibial accelerations (although non-significant) in females compared to males, with loading values between 2.6 G and 3.2 G when walking at 1.25 m/s. The greater acceleration values reported in this work may be a consequence of the higher walking speed developed in our study, and, thus, increasing the forces the participants were subjected to.

Holt et al. (2005) also aimed to compare the impact acceleration at the head and shank during walking with and without extra load in a backpack. Interestingly, they observed a reduction in tibial acceleration when a 40% load was carried, whereas values at the head were similar to those without the extra load. In contrast, Harman et al. (2000) and Goh et al. (1998) found that greater loads led to higher impact, braking and propulsive forces, although these authors did not report specific values and, therefore, the effects of backpack carrying on the force transmission was unreported. Voloshin (2000) stated accelerations measured at the tibial tuberosity to be between 1–5 G when walking and Kiskii et al. (2008) showed that the body can be subjected to accelerations over 10 G when an individual undertakes whole body vibration training. Previous research findings support the results found in this investigation, which show accelerations between 3–7 G in the shank and 2–3 G at the head.

The results found by Holt et al. (2005) show a decrease in the measured accelerations at the shank when load was added. These results are in contrast to these findings, despite a small decrease in the accelerations measured in the female subjects when a 10% weight was added, all other conditions for males and females showed minor increases in the recorded accelerations. A possible explanation for these differences is that Holt et al. (2005) constructed a rigid frame where weights were attached at shoulder height, in comparison to this study where weights were added to a backpack placed on the subjects back. As a consequence, weights were placed more in line to the lumbar area of the spine and unrestricted, allowing weights to shift during walking what may better reproduce a real-life situation.

On the other hand, Holt et al. (2005) provided evidence that the accelerations are absorbed and dissipated through the body, being greatest at the ankle and lowest at the head, what supports the findings of this study. This result is in line with the idea of other researchers who claimed that high vibration at the skull is not advantageous as it leads to a greater risk of injury, such as low back pain, sciatic pain and degenerative changes in bones ( Kiiski et al., 2008 ). Nevertheless, Holt at al. (2005) also discovered changes in the trajectory of the accelerations as a result of increased speed or load. The walking pattern changes which occur from a change in load/speed ( Harman et al., 2000 ) have an effect on the stability of the head and lead to potential falling risks. However, this study showed no or little variation in the accelerations in the head when walking with and without added load, suggesting that there was no added or abnormal movement of the head between conditions. It may be, therefore, that instability at the head is caused when excessive load is carried (greater than 30–40% of body weight), or when walking at excessive speed or on uneven terrain.

Relatively few studies have quantified the impacts on the human body. Those which have looked into forces and accelerations placed on the body typically used single sex designs ( Birrell et al., 2007; Goh et al., 1998 ; Ren et al., 2005 ). Only a minor number of studies have used mixed-sex designs, analysing males and females in the same study ( Henriksen et al., 2008 ; Holt et al., 2005 ; Keller et al., 1996 ; LaFiandra et al., 2002 ). These few studies combined results from the males and females, rather than analysing genders separately, therefore, this study is one of the first which has analysed differences between males and females. There may be many reasons why differences occurred in this study between the male and female participants. Firstly, the level of physical fitness of the participants could be a factor into different recorded maximal amplitudes in recreational activity. Kinoshita (1985) highlighted that sedentary people were more sensitive to load carriage than those familiar to carrying loads in different activities. The sports level being higher in the males compared to the females could play a role that explains why the men show lower acceleration values for every condition analysed.

Secondly, variables between the males and females need to be taken into account. Females mean height was 172 cm compared to a mean height of 179 cm in males. The backpack positioning therefore would have been in different positions and, thus, the impact loading would be at different joints ( Henriksen et al., 2008 ). As a result, the measurements recorded at the shank could vary due to the gait patterns associated with loading area and location. On the same theme of genetic makeup, males had a mean mass of 73 kg compared to females mean mass of 57 kg. As the human body acts as a natural shock absorber (bones and tissues), the greater bone and muscle masses and lengths in the males of this study compared to the females could have influenced the transmission recorded, providing more opportunity to absorb forces before they reach the head

Previous research findings that suggest a maximum of 15% BW in a backpack are supported by this study ( American Chiropractic Association , American Occupational Therapy Association , American Academy of Orthopaedic Surgeons ; Furjuoh et al., 2004 ; Hong and Li, 2005 ; Lindstrom-Hazel, 2009 ). There was an increase in the accelerations recorded on the body, both in males and females, suggesting that individuals maybe either approaching or have reached a recommended load for carriage and any greater loads may cause injury due to the forces placed on the body as well as kinematic changes in walking in order to accommodate such heavy loads ( Goh et al., 1998 ; Harman et al., 2000 ; Holt et al., 2005 ).

Future research should take into account the effects of different footwear on force transmission and absorption, effects of different walking speeds, surfaces and location of load, and the possibility of considering the effects of those with illnesses or disabilities.

The main outcome of this study shows that there are significant differences in impact accelerations between males and females when walking on a treadmill. Compared to females, both shank and head accelerations were respectively 8% and 11% lower in male participants. Even though carrying a heavier load should theoretically result in greater impact acceleration, this effect was only seen as a non-significant increase in shank acceleration values, whereas acceleration at the head remained similar regardless of the load carried. Therefore, this study provides further evidence that the human body attempts to absorb forces placed on the body in both males and females, most likely as a mechanism to protect the motor and sensory centres in the head. The mechanisms behind the gender differences are unknown and therefore, further research is needed to provide a greater understanding to this phenomenon.

## Figures and Tables

**Table 1:** *
Baseline characteristics of the participants
*

Item	Whole group (n=29)	Males (n=16)	Females (n=13)
Age (years)	24.66 ± 3.67	24.67 ± 4.38	24.28 ± 2.06
Body Height (cm)	176 ± 6.81	179 ± 4.90	172 ± 6.73
Body Mass (kg)	68.9 ± 10.27	76.09 ± 6.76	62.18 ± 8.14

*Mean ± Standard Deviation*
